# Patient choice improves self-efficacy and intention to complete tuberculosis preventive therapy in a routine HIV program setting in Uganda

**DOI:** 10.1371/journal.pone.0246113

**Published:** 2021-02-04

**Authors:** Rachel K. Lim, Fred C. Semitala, Elly Atuhumuza, Laban Sabiti, Jane Namakula-Katende, Winnie R. Muyindike, Moses R. Kamya, David Dowdy, Adithya Cattamanchi

**Affiliations:** 1 Department of Medicine, University of Calgary, Calgary, Alberta, Canada; 2 Department of Internal Medicine, Makerere University College of Medicine, Kampala, Uganda; 3 Infectious Diseases Research Collaboration, Kampala, Uganda; 4 Makerere University Joint AIDS Program, Kampala, Uganda; 5 Department of Medicine, Mbarara University of Science and Technology, Mbarara, Uganda; 6 Johns Hopkins Bloomberg School of Public Health, Baltimore, Maryland, United States of America; 7 Curry International Tuberculosis Center and Division of Pulmonary and Critical Care Medicine, University of California San Francisco, San Francisco, California, United States of America; Centro de Investigação em Saúde de Manhiça, MOZAMBIQUE

## Abstract

A 12-dose weekly regimen of rifapentine plus isoniazid (3HP) is recommended for the prevention of active tuberculosis (TB); however, it is unclear whether 3HP should be provided by directly observed therapy (DOT) or self-administered therapy (SAT). In addition, the introduction of patient informed choice between delivery modalities may have a positive impact on factors leading to treatment completion. The authors randomized 252 participants with HIV to a hypothetical scenario of providing preventive therapy by either DOT or an informed choice between DOT and SAT. Out of 104 participants who were randomized to a choice between DOT and SAT, 103 chose therapy by SAT. Participants rated their level of confidence and intention to complete therapy. Compared to those assigned to the DOT scenario, patients assigned to the choice scenario expressed greater confidence and intention to complete preventive therapy. Convenience and travel required to complete 3HP therapy were important factors in deciding between delivery modalities. Those assigned to DOT identified more barriers to completing therapy than those given a choice. Empowering patients to make informed decisions about how they receive TB preventive therapy may improve completion rates.

## Introduction

Tuberculosis (TB) is the leading cause of death among people living with HIV (PLHIV), causing approximately 250,000 deaths in 2018 [1]. It is estimated that 25% of the world’s population has latent tuberculosis infection (LTBI), and addressing this vast reservoir of infection is imperative for TB elimination [2]. The World Health Organization (WHO) strongly recommends that all PLHIV receive TB preventive therapy (TPT), which has been shown to substantially reduce the risk of TB disease [3]. A 12-week course of rifapentine and isoniazid (3HP) has been approved as a short-course TPT regimen for PLHIV. In the PREVENT TB study, 3HP was given strictly under directly observed therapy (DOT), a practice usually reserved for active TB treatment [3]. Whether DOT is beneficial in improving outcomes in active TB disease is somewhat controversial [4]. There are multiple barriers including inconvenience to the patient, long-distances, and costs associated with frequent clinic visits [5]. An open-label non-inferiority clinical trial randomized 1002 adults with LTBI to 3HP treatment by DOT or SAT in several countries and demonstrated that SAT was noninferior to DOT in terms of treatment completion in the United States, however, the pooled analysis did not meet noninferiority criteria [6]. Therefore, the optimal method of administering 3HP remains unclear.

Adherence to TPT has historically been poor, with a completion rate of only 19% reported in a review of all regimens [7]. A systematic review of factors related to patient adherence to TB treatment suggested that flexibility, choice in treatment, and efforts to encourage patient autonomy could improve adherence [8]. Within a cohort of 586 patients offered LTBI treatment over a seven-year period, Rennie and colleagues found that the odds of treatment completion increased 2.3-fold when patients were offered a choice between two LTBI treatment regimens [9]. However, it is unknown whether a choice in the modality of TPT delivery can affect treatment completion, and is often predetermined by healthcare providers in many high burden settings.

To understand whether patient choice of delivery strategy should be further investigated as a strategy to improve TPT adherence, we determined whether offering patients an informed choice between taking 3HP by DOT or self-administered therapy (SAT) could improve self-efficacy and intention scores in completing treatment relative to DOT alone. Self-efficacy and intention were chosen as the dependent variables because they are strong predictors of actual behavior according to several behavior change frameworks [10–12].

## Materials and methods

### Patient population and setting

Adults age 18 years or older with confirmed HIV were recruited during their visit to a local HIV/AIDS clinic for routine care in Kampala, Uganda between March and November 2019. This study was reviewed and approved by the University of California San Francisco Committee on Human Research, the Makerere University School of Public Health Research Ethics Committee, and the Uganda National Council for Science and Technology. All participants provided informed consent.

### Study procedures

Participants were randomized to one of two hypothetical scenarios (DOT or choice between DOT and SAT) for taking 3HP. Both hypothetical scenarios outlined that the new treatment (3HP) reduced the risk of developing active TB, the number of pills and weeks of treatment involved, and the importance of taking all prescribed doses. Participants assigned to the hypothetical DOT scenario were given a brief description of DOT. Participants assigned to the hypothetical choice scenario were given a brief description of both DOT and SAT, and a decision aid was used to help them choose their preferred mode of 3HP delivery. Following the description, all participants were read two statements in a neutral manner and asked to rate their level of agreement on a 10-point Likert scale (ranging from strongly agree to strongly disagree). The same person interacted with all participants and was not a clinician at the HIV clinic to reduce response bias. The statement to rate confidence was as follows: “I feel confident I would be capable of completing all 12 weekly doses if the treatment were offered in the manner described”. The statement to rate intention was as follows: “I would intend to complete all 12 weekly doses of the two medicines if the treatment were offered in the manner described”. Participants randomized to the choice arm were also asked about the leading factor behind their decision between delivery modalities.

### Statistical analysis

Descriptive statistics (median and IQR) were used to summarize the survey responses. Median survey responses across demographic and clinic characteristics were compared using Wilcoxon rank-sum test. Analysis was done using STATA. Participant responses on the deciding factor leading to their choice of delivery mode were recorded and analysed using grounded theory.

## Results

A total of 252 participants were enrolled. Participant demographic information is summarised in Table 1. There were 148 participants randomized to a fixed DOT scenario while 104 were randomized to an informed choice between DOT and SAT. Of those given a choice, all but one chose to receive 3HP by SAT. Compared to patients assigned to the DOT scenario, patients assigned to the choice scenario expressed higher confidence (median score 9 [IQR 9–9] vs. 7 [IQR 3–9], p<0.001) and greater intention to complete 3HP (median score 9 [IQR 9–9] vs. 8 [IQR 7–9]), p<0.001). Among patients assigned to the DOT scenario, 25% expressed low confidence (score 0–3), and 10% expressed low intention to complete 3HP. In contrast, all patients (n = 104) assigned to the patient choice scenario expressed high confidence (score 7–10) and high intention to complete 3HP. Scores did not differ across age, gender, median CD4 cell count, and viral load (Table 2).

**Table 1:** Patient demographics, N = 252.

	Delivery Strategy	
Characteristic, median (IQR)	DOT (N = 148)	Choice (N = 104)
**Age (years)**	40 (36–47)	40 (34–46)
**Male sex, no. (%)**	58 (39)	30 (29)
**Most recent CD4 (cells/mm**^**3**^**)**	453 (269–585)	449 (283–660)
**Most recent Viral Load (copies/mL)**	0 (0–0)	0 (0–20)

**Table 2:** Determinants of confidence and intention.

Variable		Confidence		Intention	
		Median (IQR)	p-value	Median (IQR)	p-value
**Treatment**	Choice	9 (8–9)	<0.001	9 (8–9)	<0.001
	DOT	7 (3–9)		8 (6–9)	
**Gender**	Male	8 (5–9)	0.093	8 (6–9)	0.067
	Female	9 (6–9)		9 (8–9)	
**Age**	Median and below	8 (6–9)	0.850	9 (7–9)	0.914
	Above median	8 (6–9)		9 (7–9)	
**CD4 count**	500 and below	8 (5–9)	0.014	8 (7–9)	0.031
	Above 500	9 (6–9)		9 (8–9)	
**Viral load**	Undetectable	8 (6–9)	0.730	8 (6–9)	0.849
	Detectable	8 (5–9)		8 (5–9)	

Qualitative analysis of 189 patient responses following the surveys explored enabling factors and barriers for completing 3HP treatment. Among those who chose 3HP by SAT, the main perceived benefit was the convenience of taking the pills at home. Most participants (108 of 189) in both groups stated that they would take 3HP because they wished to prevent TB disease. Barriers to taking therapy were self-identified within the fixed DOT group, except for one participant in the choice group who feared drug toxicity. Barriers to taking 3HP were transportation and occupational related issues. Many respondents spoke of the prohibitive cost involved with traveling to the clinic every week or the conflicts with their job. The fear of drug-related side effects was also a common barrier. Finally, a few respondents had not informed their partners of their HIV status and feared their status would be exposed if they came to the clinic every week to receive 3HP by DOT.

## Discussion

Study participants that were enabled to make an informed choice between DOT or SAT were found to have higher scores for self-efficacy and intention to complete TB preventive therapy compared to those who were assigned to a fixed DOT scenario. These findings support the importance of considering patient preferences and the impact of empowering patients to choose between treatment options when designing TPT programs in high burden countries. While numerous studies have examined the associations between sociodemographic factors, digital adherence technologies, and shorter treatment regimens on treatment completion, few studies to date have examined the impact of enabling patients to make informed choices.

Self-efficacy belief and intention were measured in this study because they are considered strong direct predictors of behavior [10–12]. Self-efficacy, which refers to a personal sense of ability to carry out a behavior, influences the degree of obstacles one is willing to overcome to reach a goal [13]. Perceived self-efficacy scales have predicted a diverse array of health behaviors including smoking cessation, physical activity, and alcohol consumption [14–17]. Molassiotis and colleagues showed that adherence to antiretroviral treatment in PLHIV was more strongly related to self-efficacy than the availability of social supports [18]. Similarly, another study demonstrated that low adherence self-efficacy was related to low medication adherence among women with HIV/AIDS [19]. Intention represents a proactive commitment to perform a future action, and although influenced by self-efficacy, is also affected by attitudes and subjective norms [10].

The vast majority of participants (103 out of 104) who were given a choice between DOT and SAT chose SAT as their preferred method of 3HP delivery, with convenience being a major influence. This is consistent with the results of a discrete choice experiment examining preferences that influence the acceptance of LTBI treatment. Guo and colleagues showed that the frequency of clinic visits was among the most important determinants of decision making, with a preference for visits every 1 or 2 months compared to every 2 weeks [20]. Our survey results likely reflect real-world choices, where patients may favor SAT (with a single check-in and refill visit) compared to weekly DOT.

This study has several limitations. First, hypothetical scenarios were used and while the results are supportive a formal randomized trial is needed to confirm whether offering patients an informed choice between delivery strategies improves treatment completion. Second, while self-efficacy and intention are established predictors of actual behavior, there may be other decisional factors including socio-economic factors that were not captured in this study. Finally, response bias is a concern in survey-based research and could have led patients to provide higher ratings of self-efficacy and intention to complete TPT. However, the bias applies to participants randomized to both arms and is therefore unlikely to impact the comparison between arms.

The Institute of Medicine defines patient-centered care as “care that is respectful and responsive to individual patient preferences, needs, and values”, and highlights it as a key component of high-quality health care [21]. We recommend that TB/HIV programs should strive to elicit and respond to patient preferences wherever possible, as there is growing evidence that this may improve the success rates of TB treatment. In addition, decision aids have been shown to facilitate informed choices that are grounded in patients’ values while increasing adherence to medical care [22]. The informed choice approach is being further investigated in the 3HP Options Implementation Trial currently underway, which will randomize PLHIV who are candidates for LTBI treatment to receive 3HP delivered by facilitated DOT, facilitated SAT, or an informed choice between facilitated DOT and SAT using a decision aid.

## Conclusion

Empowering patients to make informed decisions about TPT may positively impact treatment completion rates. Future studies should explore the impact of providing patients with an informed choice not only of delivery strategy but also daily versus weekly TPT regimens.

## References

[1] World Health Organization. Global tuberculosis report 2019. Geneva: World Health Organization; 2019 Website https://www.who.int/tb/publications/global_report/en/ Accessed March 2020.

[2] Cohen A, Mathiasen VD, Schon T, Wejse C. The global prevalence of latent tuberculosis: a systematic review and meta-analysis. Eur Respir J. 2019;54(3). 10.1183/13993003.00655-2019 31221810

[3] Latent tuberculosis infection: updated and consolidated guidelines for programmatic management. Geneva: World Health Organization; 2018 Website https://www.who.int/tb/publications/2018/latent-tuberculosis-infection/en/ Accessed March 2020. 30277688

[4] Karumbi J, Garner P. Directly observed therapy for treating tuberculosis. Cochrane Database Syst Rev. 2015(5):CD003343 10.1002/14651858.CD003343.pub4 26022367 PMC4460720

[5] Marahatta SB, Yadav RK, Giri D, Lama S, Rijal KR, Mishra SR, et al Barriers in the access, diagnosis and treatment completion for tuberculosis patients in central and western Nepal: A qualitative study among patients, community members and health care workers. PLoS One. 2020;15(1):e0227293 10.1371/journal.pone.0227293 31940375 PMC6961875

[6] Belknap R, Holland D, Feng PJ, Millet JP, Caylà JA, Martinson NA, et al Self-administered Versus Directly Observed Once-Weekly Isoniazid and Rifapentine Treatment of Latent Tuberculosis Infection: A Randomized Trial. Ann Intern Med. 2017;167(10):689–697. 10.7326/M17-1150 29114781 PMC5766341

[7] Alsdurf H, Hill PC, Matteelli A, Getahun H, Menzies D. The cascade of care in diagnosis and treatment of latent tuberculosis infection: a systematic review and meta-analysis. The Lancet Infectious Diseases. 2016;16(11):1269–78. 10.1016/S1473-3099(16)30216-X 27522233

[8] Munro SA, Lewin SA, Smith H, Engel ME, Fretheim A, Volmink J. Patient adherence to tuberculosis treatment: A systematic review of qualitative research. PLoS Med. 2007;4(7): e238 10.1371 10.1371/journal.pmed.0040238 17676945 PMC1925126

[9] Rennie TW, Bothamley GH, Engova D, Bates IP. Patient choice promotes adherence in preventive treatment for latent tuberculosis. Eur Respir J. 2007;30(4):728–735. 10.1183/09031936.00034007 17626113

[10] Ajzen I. From intentions to actions: a theory of planned behavior In: Kuhl J, Beckmann J, eds. Action Control: From Cognition to Behaviour. Berlin: Springer, 1985: pp 11–39.

[11] Bandura A. Self-efficacy: The exercise of control. New York: Freeman; 1997.

[12] Prochaska JO, Redding CA, Evers K. The Transtheoretical Model and Stages of Change In: Glanz K, Rimer BK, Lewis FM, eds. Health Behavior and Health Education: Theory, Research, and Practice (3rd Ed). San Francisco, CA: Jossey-Bass, 2002: pp 97–121.

[13] DeVellis BM, DeVellis RF. Self-efficacy and health In: Baum A, Revenson TA, Singer JE, editors. Handbook of health psychology. Mahwah, NJ: Erlbaum; 2000 p. 235–247.

[14] Feltz DL, Riessinger CA. Effects of in vivo emotive imagery and performance feedback on self-efficacy and muscular endurance. J Sport Exercise Psy. 1990;12:132–143.

[15] Christiansen M, Vik PW, Jarchow A. College student heavy drinking in social contexts versus alone. Addict Behav. 2002;27:393–404. 10.1016/s0306-4603(01)00180-0 12118627

[16] Dijkstra A, De Vries H. Self-efficacy expectations with regard to different tasks in smoking cessation. Psychology Health. 2000;15(4):501–511.

[17] Gwaltney CJ, Shiffman S, Paty JA, Liu KS, Kassel JD, Gnys M, et al (2002). Using self-efficacy judgments to predict characteristics of lapses to smoking. J Consult Clin Psychol. 2002;70:1140–1149. 10.1037//0022-006x.70.5.1140 12362964

[18] Molassiotis A, Nahas-Lopez V, Chung WY, Lam SW, Li CK, Lau TF. Factors associated with adherence to antiretroviral medication in HIV-infected patients. Int J STD AIDS. 2002;13:301–310. 10.1258/0956462021925117 11972933

[19] Murphy DA, Greenwell L, Hoffman D. Factors associated with antiretroviral adherence among HIV-infected women with children. Women Health. 2002;36:97–111. 10.1300/J013v36n01_07 12215006

[20] Guo N, Marra CA, FitzGerald JM, Elwood RK, Anis AH, Marra F. Patient preference for latent tuberculosis infection preventive treatment: a discrete choice experiment. Value Health. 2011;14(6):937–943. 10.1016/j.jval.2011.05.003 21914516

[21] Institute of Medicine (US) Committee on Quality of Health Care in America. Crossing the quality chasm: a new health system for the 21st century. Washington DC: National Academies Press, 2001. 25057539

[22] Stacey D, Legare F, Lewis K, Barry MJ, Bennett CL, Eden KB, et al Decision aids for people facing health treatment or screening decisions. Cochrane Database Syst Rev. 2017;4:CD001431 10.1002/14651858.CD001431.pub5 28402085 PMC6478132

